# Transcriptome Analysis Reveals Roles of Sucrose in Anthocyanin Accumulation in ‘Kuerle Xiangli’ (*Pyrus sinkiangensis* Yü)

**DOI:** 10.3390/genes13061064

**Published:** 2022-06-14

**Authors:** Xiangzhan Zhang, Bo Li, Ruiwei Duan, Chunhong Han, Lei Wang, Jian Yang, Long Wang, Suke Wang, Yanli Su, Huabai Xue

**Affiliations:** 1 Zhengzhou Fruit Research Institute, Chinese Academy of Agricultural Sciences, Zhengzhou 450009, China; zhangxiangzhan@caas.cn (X.Z.); nicelibo2020@163.com (B.L.); weiwei6182021@163.com (R.D.); hanchdoudou@163.com (C.H.); ewlei@163.com (L.W.); yangjian@caas.cn (J.Y.); wanglong02@caas.cn (L.W.); wangsuke@caas.cn (S.W.); suyanli@caas.cn (Y.S.); 2 Key Laboratory of Fruit Breeding Technology of Ministry of Agriculture and Rural Affairs, Zhengzhou Fruit Research Institute, Chinese Academy of Agricultural Sciences, Zhengzhou 450009, China; 3 Henan Key Laboratory of Fruit and Cucurbit Biology, Zhengzhou Fruit Research Institute, Chinese Academy of Agricultural Sciences, Zhengzhou 450009, China; 4 College of Horticulture and Plant Conservation, Henan University of Science and Technology, Luoyang 471023, China

**Keywords:** pear, anthocyanin accumulation, sucrose, coloration

## Abstract

Pear (*Pyrus* L.) is one of the most important temperate fruit crops worldwide, with considerable economic value and significant health benefits. Red-skinned pears have an attractive appearance and relatively high anthocyanin accumulation, and are especially favored by customers. Abnormal weather conditions usually reduce the coloration of red pears. The application of exogenous sucrose obviously promotes anthocyanins accumulation in ‘Kuerle Xiangli’ (*Pyrus sinkiangensis* Yü); however, the underlying molecular mechanism of sucrose-mediated fruit coloration remains largely unknown. In this study, comprehensive transcriptome analysis was performed to identify the essential regulators and pathways associated with anthocyanin accumulation. The differentially expressed genes enriched in Gene Ontology and the Kyoto Encyclopedia of Genes and Genomes items were analyzed. The transcript levels of some anthocyanin biosynthetic regulatory genes and structural genes were significantly induced by sucrose treatment. Sucrose application also stimulated the expression of some sugar transporter genes. Further RT-qPCR analysis confirmed the induction of anthocyanin biosynthetic genes. Taken together, the results revealed that sucrose promotes pear coloration most likely by regulating sugar metabolism and anthocyanin biosynthesis, and this study provides a comprehensive understanding of the complex molecular mechanisms underlying the coloration of red-skinned pear.

## 1. Introduction

Pear (*Pyrus* L.) is one of the most important fruit crops and is widely cultivated worldwide. It has a history of more than 3000 years in China [1]. The annual production of pear in China reached 16.10 million tons and accounted for about 70% of the global total in 2020 (https://www.fao.org/faostat/en/#data/QI, accessed on 15 October 2021). Compared with traditional green-yellow or russet pears, red pears generally exhibit an attractive appearance and appeal to consumers’ preferences [2,3].

Red-skinned pears are a result of the accumulation of anthocyanins in the peel. Anthocyanins are important components of water-soluble flavonoids that are responsible for the pigmentation of plant leaves, flowers, fruits, petals, stems, seeds, and other tissues [4]. The accumulation of anthocyanins is beneficial for pollinator attraction, seed dispersal, defense against pathogens and bacteria, biotic and abiotic stress resistance, and ultraviolet protection [5]. Moreover, anthocyanins act as antioxidant molecules that contribute to the scavenging of free radicals and are beneficial for the human body, e.g. in the prevention of cardiovascular diseases and heart disease [6,7,8,9,10]. As the most widely accepted compounds in fruits, flavonoids increase human dietary benefits [11].

The accumulation of anthocyanins is affected by internal and external stimuli, and genetic factors play dominant roles during this process [12]. The anthocyanin biosynthesis pathway has been extensively investigated, and it is well characterized that the biosynthesis of anthocyanins is regulated by a cascade of coordinated enzymes, including phenylalanine ammonia-lyase (PAL), cinnamate 4-hydroxylase (C4H), 4-coumarate:coenzyme A ligase (4CL), chalcone synthase (CHS), chalcone isomerase (CHI), flavanone 3-hydroxylase (F3H), flavonoid 3′-hydroxylase (F3′H), flavonoid 3′,5′-hydroxylase (F3′5′H), dihydroflavonol 4-reductase (DFR), anthocyanin synthase (ANS), and flavonoid 3-*O*-glucosyltransferase (UFGT) [12,13]. Furthermore, the accumulation of anthocyanins is also associated with the transport of anthocyanins in plants. Three main pathways are reported to be involved in this transport process: the glutathione *S*-transferase (GST) family [14], the ATP-binding cassette (ABC) family, and the multidrug and toxic compound extrusion (MATE) family [15,16].

Apart from structural genes, the biosynthesis of anthocyanins is regulated by regulatory genes, among which the MYB-bHLH-WD40 (MBW) complex has been extensively reported [17,18,19]. The crucial role of the MYB transcription factor in anthocyanin accumulation was fully documented in diverse plant species, including *Arabidopsis*, apple, grape, and strawberry [20,21]. In addition to the MBW complex, other types of regulatory proteins were also reported to be involved in anthocyanin biosynthesis, including members of the NAC transcription family [22], WRKY transcription factors [23], ERF transcription factor family [24], bZIP transcription factors [25], MADS-box proteins [26], SPL transcription factors [27], BBX proteins [28], and other regulatory genes. These transcription factors have positive or negative roles in regulating the expression of structural anthocyanin biosynthesis genes and determine anthocyanin accumulation under developmental and environmental regulation [13].

External environmental factors, including light, UV-B radiation, low temperature, phytohormones, and the application of exogenous substances, usually modulate the biosynthesis of anthocyanins, which may be associated with the biosynthesis of substrates for metabolism in the processes of anthocyanin formation [13,29].

‘Kuerle Xiangli’ (*P. sinkiangensis*), a native landrace in the Xinjiang Autonomous Region of China, has long been favored by consumers for its juicy flesh, crisp texture, distinct fragrance, and red skin. However, the red skin of ‘Kuerle Xiangli’ is usually impaired by the occurrence of extreme environmental conditions and early harvesting, which significantly reduce the commercial value to a certain extent. The application of exogenous sucrose could improve the coloration of ‘Kuerle Xiangli’. However, the underlying molecular mechanism of the sucrose and signal transduction of anthocyanin synthesis has not been explored. In this study, comprehensive physiological and transcriptional analyses were performed on ‘Kuerle Xiangli’ to reveal the possible pathways and differentially expressed genes (DEGs) involved in sucrose-mediated anthocyanin accumulation, which may provide insight into anthocyanin accumulation and the application of sucrose in production to improve fruit coloration.

## 2. Results

### 2.1. Sucrose Application Enhanced Anthocyanin Accumulation in ‘Kuerle Xiangli’

**Figure 1:**
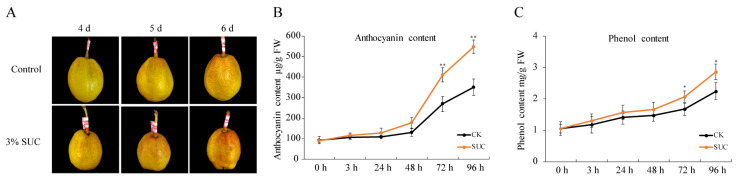
Application of exogenous sucrose promoted anthocyanin accumulation in ‘Kuerle Xiangli’. (**A**) Phenotypes of ‘Kuerle Xiangli’ under sucrose treatment at different time points. Measurement of the (**B**) anthocyanins and (**C**) phenol content in ‘Kuerle Xiangli’ under sucrose treatment. Asterisks indicate statistical significance (*, *p* < 0.05; and **, *p* < 0.01) calculated by Student’s *t*-test.

To explore the possible exogenous substance to improve the coloration of ‘Kuerle Xiangli’, a set of exogenous applications was investigated. Green ‘Kuerle Xiangli’ pears in the absence of direct sunlight were sampled at maturity and were then treated with or without 3.0% (*w*/*v*) sucrose at 17 °C under continuous light and 80% humidity in a light incubator. After treatment for three days, the peels accumulated more anthocyanins than the corresponding controls without sucrose treatment. The measurement of the total anthocyanins and the phenol content showed that pears subjected to sucrose treatment had more anthocyanins and phenol (Figure 1), indicating that the application of sucrose enhanced anthocyanin accumulation in ‘Kuerle Xiangli’.

### 2.2. Analysis of Pear Transcriptome following Sucrose Treatment

To investigate the transcriptional processes of the sucrose-induced fruit coloration, RNA-seq was performed with ‘Kuerle Xiangli’ treated with 3.0% (*w*/*v*) sucrose at four time points (0 h, 3 h, 24 h, and 72 h) with three biological repeats. A total of 49.6 Gb clean data were obtained from 12 paired-end libraries, with an average of 4.13 Gb data in each sample. The Q20 value of the tested samples ranged from 98.00% to 98.27%, and the Q30 value ranged from 93.70% to 94.41%, indicating the high quality of the reads. The *P. communis* Bartlett DH genome was used as the reference genome; the average mapping rate reached 86.52%, and the average GC content was 45.98% (Table 1). A total of 40,747 genes were identified, and 18,898 genes had an FPKM value higher than 1.

**Figure 2:**
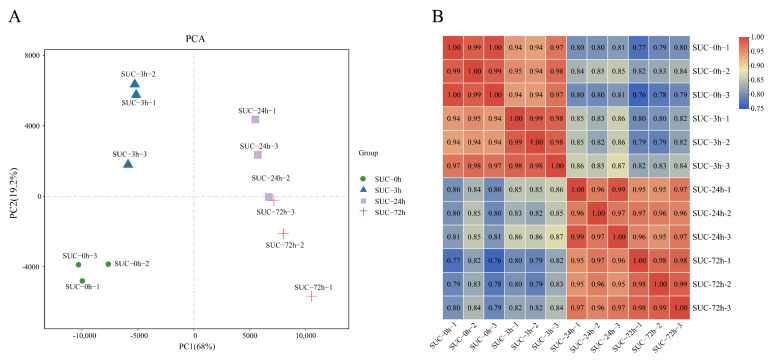
Correlation analysis of samples under sucrose treatment at different time points. (**A**) Principal component analysis (PCA) of samples subjected to sucrose treatment at different time points. (**B**) Pearson’s correlation coefficient analysis of the 12 samples under sucrose treatment at indicated time points. The red scale indicates a high correlation, and the blue scale indicates a low correlation.

Principal component analysis (PCA) was performed to analyze the relationships between the samples in the three biological repeats as well as among the different groups. The results indicated that the samples belonging to the same biological replicates at each time point mostly clustered together, indicating the good consistency of the samples within replicates at different time points (Figure 2A). Pearson’s correlation coefficient analysis indicated the positive correlation of the samples, which indicates the reliability of the experiment (Figure 2B).

**Figure 3:**
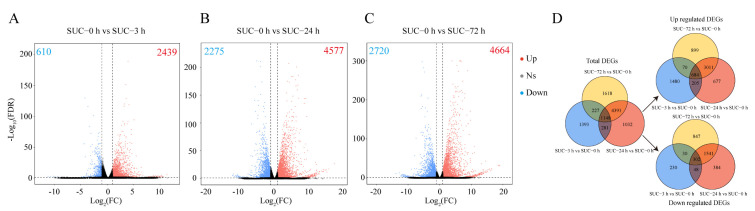
Analysis of DEGs in different groups. (**A**) Volcano plot of DEGs in response to sucrose treatment for 0 h vs. 3 h. (**B**) Volcano plot of DEGs in response to sucrose treatment for 0 h vs. 24 h. (**C**) Volcano plot of DEGs in response to sucrose treatment for 0 h vs. 72 h. (**D**) Venn diagram of the DEGs in different groups under sucrose treatment. Red dots indicate upregulated genes, and blue dots indicate downregulated genes. Gray dots indicate no significant differentially expressed genes. Ns, no significant difference. The numbers of upregulated and downregulated DEGs are shown in the corresponding figures.

Based on the RNA-seq data, genes with FDR ≤ 0.05 and |log2FC| ≥ 1 were identified as differentially expressed genes (DEGs). A total of 2439 upregulated DEGs and 610 downregulated DEGs were identified under sucrose treatment for 0 h vs. 3 h (Figure 3A); 4577 upregulated DEGs and 2275 downregulated DEGs were identified under sucrose treatment for 0 h vs. 24 h (Figure 3B); and 4664 upregulated DEGs and 2720 downregulated DEGs were identified under sucrose treatment for 0 h vs. 72 h (Figure 3C). In total, 1148 DEGs were identified and shared among these groups, of which 684 and 302 DEGs were constantly up- or downregulated (Figure 3D). It was clearly revealed that sucrose treatment upregulated more genes in pear peels in different groups. The DEGs in other groups were also analyzed, and 6313, 7444, and 957 DEGs were identified when subjected to sucrose application for 3 h vs. 24 h, 3 h vs. 72 h, and 24 h vs. 72 h, respectively (Figure S1). The results suggested that the transcript level of many genes was changed by sucrose treatment.

### 2.3. Gene Ontology (GO) Enrichment Analysis of DEGs under Sucrose Treatment

**Figure 4:**
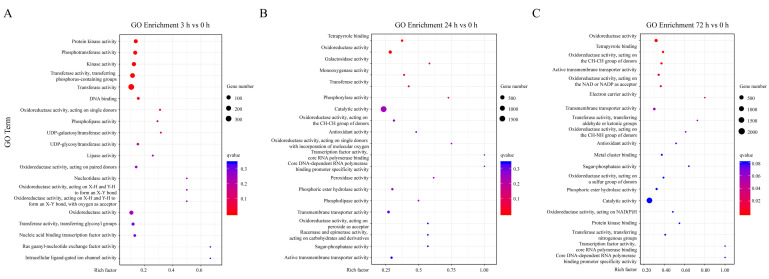
GO enrichment analysis of DEGs in different groups. (**A**) GO enrichment analysis of the DEGs in response to sucrose treatment for 3 h and 0 h. (**B**) GO enrichment analysis of the DEGs in response to sucrose treatment for 24 h and 0 h. (**C**) GO enrichment analysis of the DEGs in response to sucrose treatment for 72 h and 0 h. The dots indicate the number of DEGs, and the color scale indicates the *q*-value.

To further investigate the possible molecular functions of the DEGs in different groups, GO enrichment analysis was performed. The results of the GO terms under sucrose treatment for 0 h and 3 h indicated that the DEGs were significantly enriched in terms of basic molecular functions, including ‘Protein kinase activity’, ‘Phosphotransferase activity’, ‘Transferase activity’, ‘DNA binding’, and ‘Oxidoreductase activity’. In addition, the categories including ‘UDP-galactosyltransferase activity’ and ‘UDP-glycosyltransferase activity’ were also highly enriched, which were possibly associated with the function of sucrose in anthocyanin accumulation (Figure 4A). The GO terms under sucrose treatment for 24 h and 72 h compared with 0 h were also analyzed (Figure 4B,C). The results indicated that the two groups exhibited similar enriched terms, including ‘Tetrapyrrole binding’, ‘Oxidoreductase activity’, ‘Galactosidase activity’, ‘Monooxygenase activity’, ‘Transferase activity’, ‘Phosphorylase activity’, ‘Catalytic activity’, ‘Transmembrane transporter activity’, and ‘Sugar-phosphatase activity’, indicating the DEGs were significantly involved in these biological processes.

Comprehensive GO analysis was also performed for other comparisons, including sucrose treatment for 3 h vs. 24 h, 3 h vs. 72 h, and 24 h vs. 72 h. Significant GO terms were enriched in different groups, including, ‘Tetrapyrrole binding’, ‘Oxidoreductase activity’, ‘Sugar-phosphatase activity’, ‘Catalytic activity’, ‘Kinase activity’, ‘Galactosyltransferase activity’, ‘Transcription factor activity’, ‘Monooxygenase activity’, and ‘Galactosidase activity’ (Figure S2).

### 2.4. KEGG Enrichment Analysis of DEGs under Sucrose Treatment

**Figure 5:**
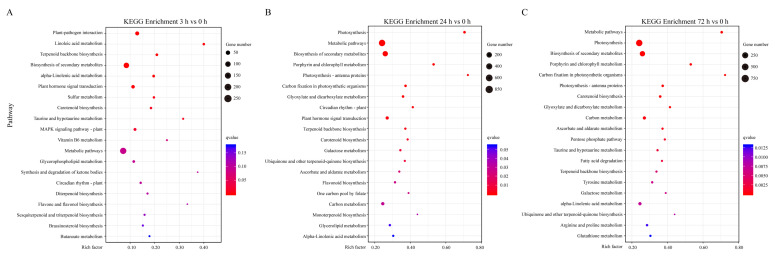
KEGG enrichment analysis of DEGs in different groups. (**A**) KEGG enrichment analysis of the DEGs in response to sucrose treatment for 3 h and 0 h. (**B**) KEGG enrichment analysis of the DEGs in response to sucrose treatment for 24 h and 0 h. (**C**) KEGG enrichment analysis of the DEGs in response to sucrose treatment for 72 h and 0 h.

To investigate the biological functions of the DEGs mediated by sucrose treatment in different groups, KEGG enrichment analysis was performed. The KEGG enrichment analysis from different groups indicated that the DEGs were significantly enriched in a numbers of metabolic pathways, which were shared, including ‘Biosynthesis of secondary metabolites’, ‘Metabolic pathways’, ‘Carotenoid biosynthesis’, ‘Terpenoid backbone biosynthesis’ and ‘Flavone and flavonol biosynthesis’, indicating that the sucrose-mediated DEGs participated in these pathways, especially the ‘Flavone and flavonol biosynthesis’ and ‘Carotenoid biosynthesis’ pathways (Figure 5).

Further analysis also revealed that at the early stage of sucrose treatment (SUC-0 h vs. SUC-3 h), specific enriched pathways were observed, including ‘Plant hormone signal transduction’, ‘Plant–pathogen interaction’, ‘Synthesis and degradation of ketone bodies’, ‘Glycerophospholipid metabolism’, and other metabolic pathways, indicating an early response to sucrose treatment (Figure 5A). At the middle (SUC-0 h vs. SUC-24 h) and later stages (SUC-0 h vs. SUC-72 h) of sucrose treatment, unique enriched pathways were detected, including ‘Porphyrin and chlorophyll metabolism’, ‘Galactose metabolism’, ‘Ubiquinone and other terpenoid–quinone biosynthesis’, and ‘Glyoxylate and dicarboxylate metabolism’ (Figure 5B,C), indicating the possible involvement of DEGs in these pathways during these periods.

KEGG enrichment analysis was also performed in other groups, including SUC-3 h vs. SUC-24 h, SUC-3 h vs. SUC-72 h, and SUC-24 h vs. SUC-72 h. The results indicated that the DEGs in these groups exhibited similar and common metabolic pathways, and the pathways, including ‘Carotenoid biosynthesis’, ‘Terpenoid backbone biosynthesis’, and ‘Flavone and flavonol biosynthesis’ were observed in different groups, indicating that sucrose treatment was involved in these processes (Figure S3).

### 2.5. Analysis of Anthocyanin Biosynthetic Structural Genes under Sucrose Treatment

**Figure 6:**
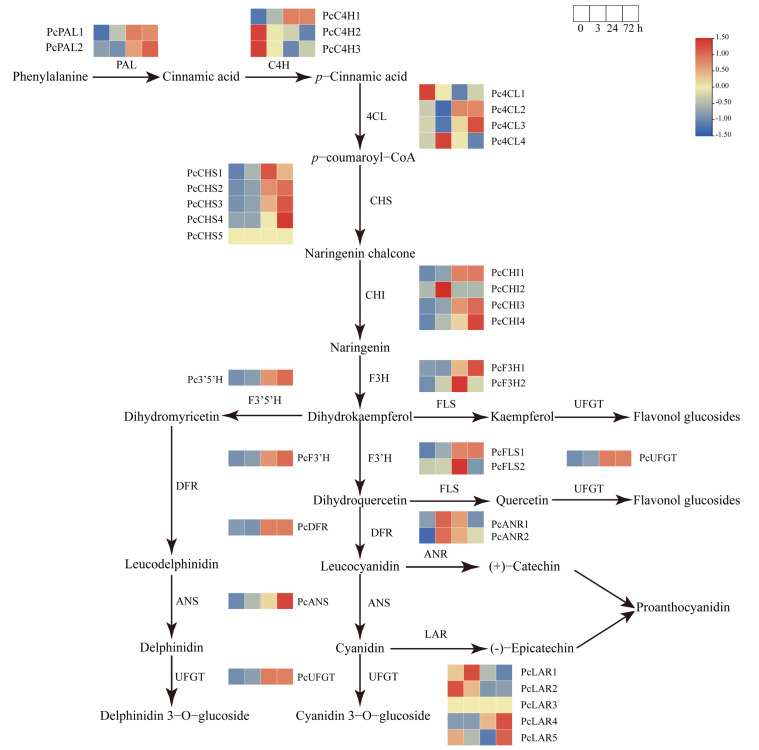
Heatmap of the expression profiles of structural genes involved in anthocyanin biosynthesis pathways under sucrose treatment. The color scale indicates the gene expression levels. Red indicates a high expression level and green indicates a low expression level.

The biosynthesis of anthocyanin is regulated by multiple structural genes that encode corresponding catalytic enzymes in the phenylpropanoid pathway. To better understand the underlying mechanisms of sucrose-induced anthocyanin accumulation in pear, the well-annotated phenylpropanoid pathway involved in anthocyanin biosynthesis was analyzed using the RNA-seq data. A total of 39 structural genes encoding 14 key enzymes involved in anthocyanin biosynthetic pathway were identified based on the RNA-seq data (Figure 6), including *PAL*, *C4H*, *4CL*, *CHI*, *CHS*, *F3H*, *F3′H*, *DFR*, *ANS*, *UFGT*, *FLS*, *F3′5′H*, *LAR*, and *ANR* (Table S1).

The results revealed that the transcript levels of some structural genes at the earlier stage of anthocyanin biosynthesis, including members of *PcPALs*, *PcC4Hs*, and *Pc4CLs*, exhibited induced transcript levels under sucrose treatment. However, the transcription of some members, such as *PcC4H2*, *PcC4H3*, *Pc4CL1*, and *Pc4CL3*, showed no obvious change when subjected to sucrose, indicating their diverse roles at the early stage of the phenylpropanoid pathway. The structural genes at the middle stage of anthocyanin biosynthesis, including most members of *PcCHSs*, *PcCHIs*, *PcF3Hs*, *PcF3′H*, *PcF3′5′H*, and *PcFLSs*, exhibited similar and significantly upregulated transcript levels under sucrose treatment (Figure 6), indicating their possible roles in sucrose-mediated anthocyanin biosynthesis. The structural genes at the later stage of anthocyanin biosynthesis, including most members of *PcCHSs*, *PcDFR*, *PcANSs*, and *PcUFGTs*, exhibited obvious upregulated and similar transcript patterns in response to sucrose treatment (Figure 6). Significant transcript changes were detected at the time points of 24 h and 72 h, suggesting the possible role of sucrose involved in anthocyanin biosynthesis.

The results also revealed that although some structural genes were involved in the flavonoid biosynthesis pathway, such as *PcLARs* and *PcANRs*, their transcription patterns were different (Figure 6), suggesting their distinct roles under sucrose treatment. This may be because *PcLARs* and *PcANRs* are responsible for the biosynthesis of proanthocyanin rather than anthocyanin. Further investigation needs to be performed to reveal the possible reason. Above all, the result indicated that sucrose treatment could induce the expression of most structural genes involved in anthocyanin biosynthesis.

### 2.6. Analysis of Anthocyanin Biosynthetic Regulatory Genes under Sucrose Treatment

**Figure 7:**
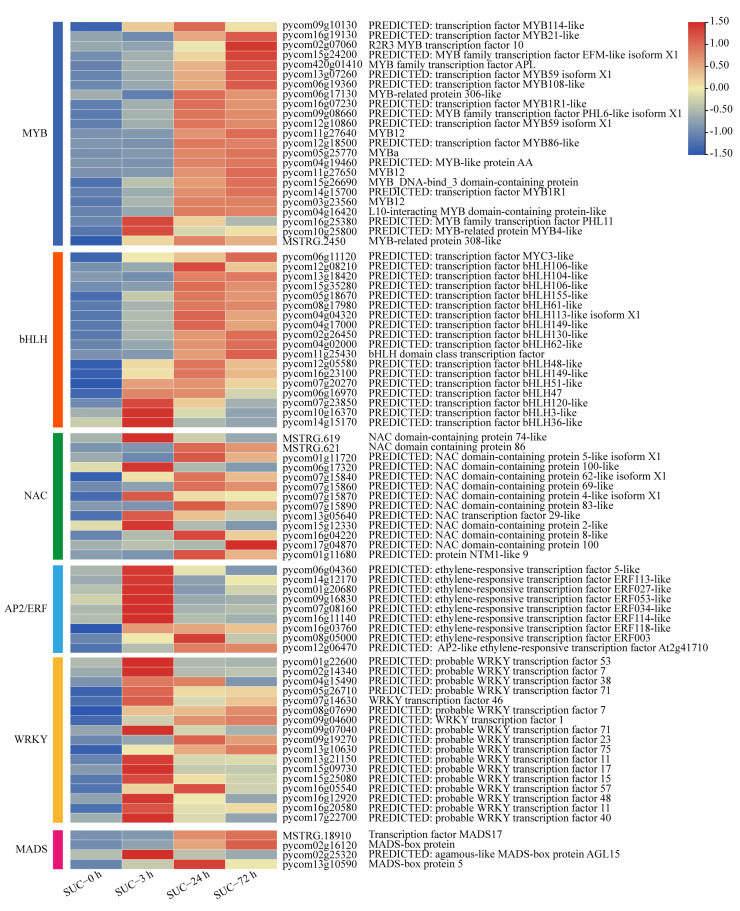
The expression profiles of different transcription factor families under sucrose treatment. Heatmap of the expression levels of sugar transporter genes using the TBtools with the data normalized to log scale and row scale. Red indicates high expression level and blue indicates low expression level.

An increasing number of studies indicate that anthocyanin biosynthesis is regulated by different transcription factors in diverse plant species [11,12,13]. To investigate the potential transcription factors involved in anthocyanin biosynthesis under sucrose treatment, the transcript levels of a set of transcription factors, including MYBs, bHLHs, NACs, AP2/ERFs, WRKYs, and MADSs, were analyzed at different time points (Table S2). The results indicated that many MYB transcription factors were significantly induced by sucrose treatment, including the MYB genes annotated with *MYB114*, *MYB10*, and other members of *MYBs*, especially at the time points of 24 h and 72 h (Figure 7), indicating the possible role of these MYB genes involved in sucrose-mediated pear coloration. The bHLH family was analyzed, and a total of 18 bHLH genes exhibited significant upregulation when subjected to sucrose treatment, including *bHLH106*, *bHLH104*, *bHLH155*, and other *bHLHs*, among which *bHLH61* (pycom08g17980) was mostly induced with more than six folds (Figure 7). Nine ERFs, 13 NACs, 17 WRKYs, and four MADSs showed obvious induction when subjected to sucrose treatment (Figure 7), indicating the possible involvement of these transcription factors in the sucrose-meditated anthocyanin accumulation.

### 2.7. Analysis of Sugar Transporter Genes under Sucrose Treatment

**Figure 8:**
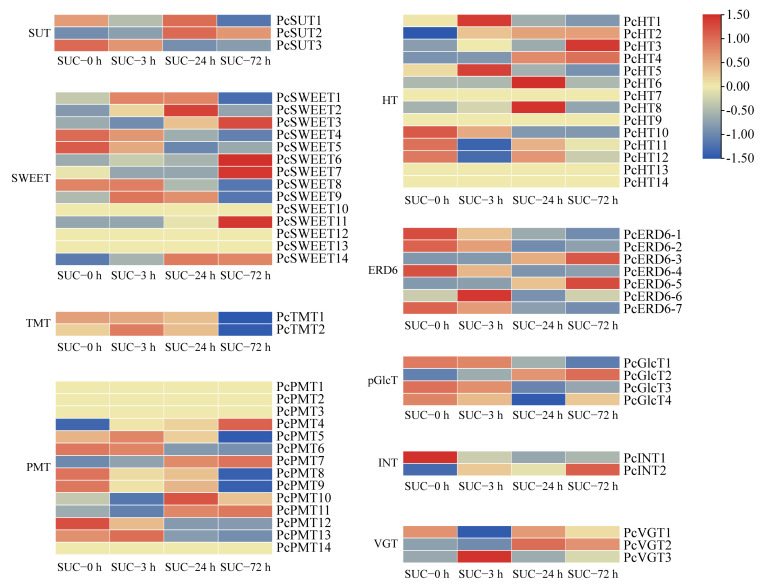
The expression profiles of the sugar transporter genes under sucrose treatment. Heatmap of the expression levels of sugar transporter genes using TBtools with the data normalized to a log scale and row scale. Red indicates a high expression level and green indicates a low expression level.

To investigate the effect of sucrose on the transport of sugars, the transcription patterns of sugar transporters, including the sucrose transporter (SUT), sugars that will eventually be exported transporters (SWEET), the tonoplast monosaccharide transporter (TMT), polyol monosaccharide transporter (PMT), hexose transporter (HT), early response to dehydration 6 (ERD6), plastidic glucose translocator (pGlcT), inositol transporter (INT), and vacuolar glucose transporter (VGT) were analyzed. Three SUT genes, 14 SWEET genes, two TMT genes, 14 PMT genes, 14 HT genes, seven ERD6 genes, four pGlcT genes, two INT genes, and three VGT genes were identified by comprehensive genome analysis (Table S3). Most members of SWEETs showed upregulated expression patterns when subjected to sucrose treatment. However, the transcript levels of more than half of the other sugar transporter genes were downregulated, especially members in the TMT and pGlcT families (Figure 8). Some sugar transporters showed no close relationship with sucrose treatment, such as PcPMT8/9 and PcHT11/12, which exhibited no regular changes under sucrose treatment (Figure 8). The application of exogenous sucrose can affect the expression of sugar transporters, implying that sucrose treatment may regulate the transport of sugars, which may be the reason for the coloration of the pear peels.

### 2.8. WGCNA of Critical Genes Involved in Sucrose-Mediated Coloration

**Figure 9:**
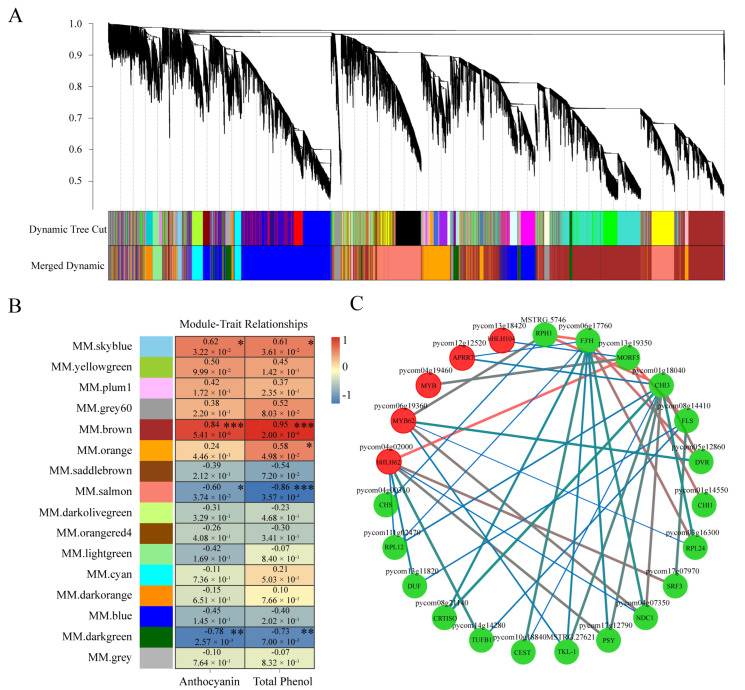
WGCNA of differentially expressed genes under sucrose treatment. (**A**) Hierarchical cluster analysis of the non-redundant DEGs involved in 16 modules. The co-expressed DEGs were exhibited at the end of the branches in the hierarchical cluster tree, and the modules were represented by designated colors. (**B**) Correlation analysis of the modules and the total anthocyanin and phenol content. The panel with designated colors indicates different modules. The color scale represents the correlations between different modules and corresponding traits. The upper data in each box indicate the value of correlations and the data below indicate the *p*-value. Asterisks indicate statistical significance (*, *p* < 0.05; **, *p* < 0.01, ***, *p* < 0.001). (**C**) Cytoscape analysis of the co-expressed DEGs in the module of “MM. skyblue”. The dots with red and green colors indicate the regulatory genes and structural genes, respectively. The red and blue lines indicate higher and lower weight value, respectively.

The accumulation of anthocyanin was significantly induced by sucrose treatment. To identify the critical genes involved in sucrose-mediated anthocyanin accumulation, weighted gene co-expression network analysis (WGCNA) was performed with the non-redundant DEGs. The results show that these DEGs could be classified into 16 modules, which are represented by the indicated color scales (Figure 9A,B). Each module contained genes with highly correlated transcription patterns. According to the analysis of the module–trait relationships, the “MM. brown” module was highly correlated with the total anthocyanins and phenol content (r = 0.84, *p* = 5.41 × 10^−4^ and r = 0.95, *p* = 2.00 × 10^−6^, respectively) and followed by the “MM. skyblue” module (r = 0.62, *p* = 3.22 × 10^−2^ and r = 0.61, *p* = 3.61 × 10^−2^, respectively) (Figure 9B).

Specifically, the “MM. skyblue” module contained most anthocyanin biosynthetic genes, including 19 structural genes of *PAL*, *4CL*, *CHS*, *CHI*, *F3H*, *F3′H*, *F3′5′H*, *DFR*, *ANS*, *UFGT*, and *FLS* (Table S1). The “MM. brown” module contained four structural genes associated with anthocyanin accumulation, including *ANR*, *4CL*, and two *CHI-like* genes (Table S1). In our database, 16 genes annotated with MYB transcription factors were identified in the module of “MM. skyblue”, including the mostly reported *PcMYB114* and *PcMYB10*, and only two MYB genes, ‘*MYB4*’ and ‘*PHL11*’, were classified into the “MM. brown” module. Further, a regulatory network containing MYBs, bHLHs, and anthocyanin biosynthetic structural genes in the module of “MM. skyblue” was analyzed, indicating the possible relationship of the regulatory genes and structural genes involved in sucrose-induced anthocyanin accumulation (Figure 9C).

Interestingly, apart from bHLH3, most sucrose-induced AP2/ERF and WRKY transcription factors also in this module were grouped into the module of “MM. brown”, implying the possible function of these genes involved in the sucrose-mediated coloration (Table S2).

### 2.9. RT-qPCR Analysis of Transcript Level of Several Anthocyanin Biosynthetic Genes

**Figure 10:**
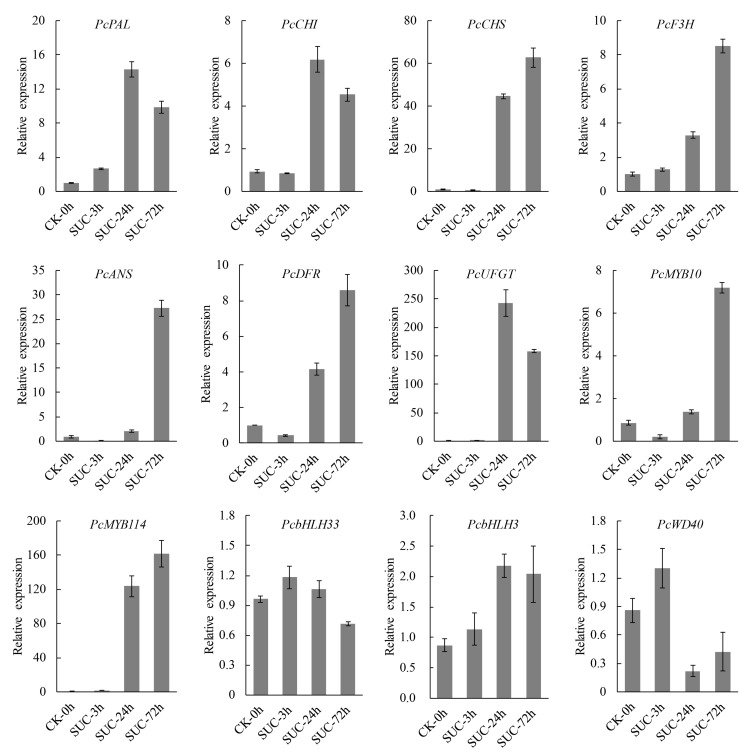
RT-qPCR analysis of anthocyanin biosynthesis-related structural and regulatory genes under sucrose treatment. Error bars indicate the mean ± SD of three independent replicates.

To verify the expression of the anthocyanin biosynthetic genes obtained from the RNA-seq data, RT-qPCR analysis was performed. Several structural genes involved in anthocyanin biosynthesis genes including *PAL*, *CHS*, *CHI*, *F3H*, *ANS*, *DFR*, and *UFGT* were selected for RT-qPCR analysis under sucrose treatment. The results indicated that the transcript levels of the above structural genes were significantly induced under sucrose treatment at different time points, especially at 24 h and 72 h. The transcript levels of the tested structural genes were induced more than fivefold, and the expression of *UFGT* was induced more than 200-fold. Furthermore, the transcript levels of several regulatory genes involved in anthocyanin biosynthesis were analyzed, including *MYB10*, *MYB114*, *bHLH3*, *bHLH33*, and *WD40*. The results showed that *MYB10* and *MYB114* were significantly induced by sucrose treatment, and the expression of *MYB114* was induced more than 100-fold. The expressions of *bHLH3*, *bHLH33*, and *WD40* were not induced or were slightly induced by sucrose treatment. The transcript levels of the tested genes showed similar consistency between RNA-seq data and RT-qPCR analysis (R^2^ = 0.82) (Figure S4), indicating the reliability of the RNA-seq data. Taken together, the results suggested that the sucrose-mediated fruit coloration in ‘Kuerle Xiangli’ was due to the upregulation of the expression of the structural genes and regulatory genes related to anthocyanin biosynthesis (Figure 10).

## 3. Discussion

Anthocyanin accumulation is responsible for the coloration of many fruit species, including apple, peach, and pear. The accumulation of anthocyanin is regulated by diverse factors, including internal regulatory and structural genes, environmental factors, and exogenous substances. Previous study revealed that the application of exogenous glutamic acid enhanced coloration in apple, peach leaves, and pear. Furthermore, the application of sucrose improved anthocyanin accumulation in *Arabidopsis*, strawberry, and radish [30,31]. In this study, the application of sucrose effectively enhanced the accumulation of anthocyanin in the peels of ‘Kuerle Xiangli’ (Figure 1), demonstrating that sucrose contributes to fruit coloration in pear.

As a basic substance, sucrose is produced in various plant species and regulates diverse metabolic and developmental processes, including carbohydrate metabolism, cotyledon development, light signaling, cell division, tuber development, floral induction, sucrose transporters, and various other biological processes. There is also evidence that sucrose induces anthocyanin accumulation in *Arabidopsis* and pear leaves [29,32,33]. Moreover, sucrose is more efficient than glucose and other exogenous substances in promoting anthocyanin accumulation in *Arabidopsis* and strawberry [32,34]. Our research demonstrated that sucrose is more effective than exogenous glucose and fructose in inducing pear coloration (Figure S5), indicating the specific role of sucrose in anthocyanin accumulation, rather than other sugars.

Traditionally, sucrose has been viewed as main source for energy supply in plants [30]. Sucrose can be hydrolyzed to other kinds of sugars, including fructose, glucose, and trehalose 6-phosphate [30,35]. Sucrose provides substrates for the biosynthesis of basic substances for plant development, including starch, cellulose, callose, and other cellular components [36]. Recently, sucrose was also considered to be a signal in the regulation of fruit ripening and other sucrose-specific biological processes [37,38,39,40]. Our RNA-seq analysis revealed that the DEGs were enriched in carbon metabolism terms, including ‘Phosphotransferase activity’, ‘Catalytic activity’, ‘Sugar-phosphatase activity’, and ‘Oxidoreductase activity’ (Figure 4). The corresponding KEGG analysis revealed that the DEGs were enriched in the ‘Glyoxylate and dicarboxylate metabolism’, ‘Galactose metabolism’, ‘Carbon metabolism’, ‘Pentose phosphate pathway’, and ‘Fatty acid degradation’ pathways (Figure 5), which are associated with the formation of carbon skeletons in plants. It is possible that sucrose may provide a basic source or substrate for anthocyanin biosynthesis or may serve as a signal for anthocyanin induction. However, further research needs to be performed to verify the possible inference.

Evidence revealed that sucrose affects phosphatase/dephosphatase activity in plants. The phosphorylation of H^+^-ATPase relies on the accumulation of sucrose under high light conditions, indicating the tight relationship between phosphorylation and sucrose treatment [41]. MdMYB1 is phosphorylated by MdMPK4 to enhance its stability under light conditions and improve fruit coloration in apple. The MAPK cascade regulates anthocyanin biosynthesis by sucrose-mediated signaling in *Arabidopsis thaliana* [42]. In our research, the GO enrichment analysis revealed that the DEGs were significantly enriched in the GO terms ‘Protein kinase activity’, ‘Phosphotransferase activity’, and ‘Phosphorylase activity’ (Figure 4), indicting the possible role of sucrose in protein phosphorylation and anthocyanin accumulation. The details of the phosphorylation process and sucrose-mediated fruit coloration in pear need to be further explored.

Evidence indicates that sucrose, rather than other sugars, regulates starch biosynthesis in the process of carbohydrate metabolism in different plant species [43,44,45]. Our KEGG enrichment analysis showed that the DEGs were significantly enriched in the pathway (Figure 5), which is consistent with previous research, indicating that the application of exogenous sucrose may contribute to the biosynthesis of starch.

A previous study indicated that sucrose engages in crosstalk with plant hormones, such as abscisic acid (ABA), jasmonate (JA), auxin, and gibberellic acid (GA) [35,46,47]. GA represses sucrose-mediated anthocyanin accumulation in *Arabidopsis* seedlings, whereas JA and ABA have a synergic effect with sucrose in anthocyanin accumulation. The application of exogenous sucrose promoted ABA accumulation and accelerated coloration and ripening in strawberry [34,35]. Our analysis indicated that the KEGG ‘Plant hormone signal transduction’ pathway was significantly enriched (Figure 5). However, it is not clear whether sucrose and other plant hormones act together to mediate fruit coloration, especially in climactic and non-climacteric fruits.

The exogenous application of sucrose affects sucrose transport in the phloem vascular tissues, which may be responsible for the fruit flavor and quality improvement during fruit maturation [35,48]. Sucrose acts as a principal form of carbon transport from the source to other organs in diverse plant species [49]. The transport of sucrose occurs throughout different cell organelles in plants, including the influx of sucrose through the plasma membrane, across the symplast, via the endomembranes and across the apoplast or via the DELLA protein [50]. It was also revealed that the transport of sucrose in plant cells requires facilitators owing to its polar characteristic and large molecular weight, and the transport may require energy or be passive [49]. Our study suggested that the genes encoding sucrose and hexose transporters were significantly affected after sucrose application, including SUTs and SWEETs (Figure 8), which is consistent with a previous study on *Arabidopsis* guard cells [51], indicating that the application of sucrose promoted the sucrose transport in plant cells. A previous study revealed that the members of the SUT gene family in various plant species exhibited different transcript patterns, and their corresponding encoded proteins have distinct roles and subcellular localizations [49]. In addition, not all of the transcript levels of the sugar transporter genes were regulated by the sucrose treatment, and our results suggested that several members of the sugar transporters were not affected by sucrose treatment, including *PcSUT1*, *PcINT*, and other genes (Figure 8).

In this study, a cascade of regulatory genes including MYB, bHLH, NAC, WRKY, and ERF transcription factors were significantly induced by sucrose treatment, and their orthologs in other plant species associated with anthocyanin accumulation were analyzed. For instance, for the MYB transcription factor in pear with the gene ID of pycom05g25770, the highest hit score in apple was MD05G1276500, which was reported to be involved in anthocyanin accumulation [52]. Moreover, pycom05g25770 was closely related to *PpMYB114*, which was responsible for the red coloration in the pear ‘Bayuehong’, indicating a similar function of pycom05g25770 in the regulation of fruit coloration [18]. For bHLH transcription factors, pycom15g35280 and pycom08g17980, which were homologous to *PpbHLH64* (Accession No. Pbr001646.1), significantly induced anthocyanin accumulation in Asian pear [19]. Furthermore, *MdbHLH3* (Accession No. MD11G1286900) promoted fruit coloration in response to low temperatures in apple, and its ortholog pycom11g25430 in pear exhibited upregulated expression levels when subjected to sucrose treatment [53]. In addition, NAC [22,54], WRKY [55,56], ERF [57,58], and MADS-box [59,60], transcription factors associated with anthocyanin accumulation, also had corresponding orthologs in pear in our research (Figure 7). These results indicated that these transcription factors are involved in sucrose-mediated anthocyanin accumulation. However, how these transcription factors in response to the sucrose signal in pear and the precise regulatory network remains to be further investigated.

## 4. Materials and Methods

### 4.1. Plant Materials and Experimental Treatments

Green ‘Kuerle Xiangli’ (*P. sinkiangensis*) fruits grown in the absence of direct sunlight were sampled from 7-year-old trees planted in an orchard in Korla (Xinjiang, China). The fruits were treated with sterile distilled water or a solution of 3.0% sucrose (g/L) mixed with Tween-20 (0.01% *v*/*v*) as a nonionic surfactant, and were then transferred to a light incubator for continuous light for three days. The conditions were as follows: 33,000 lux light intensity and 80% relative humidity at 17 °C, as described previously [14].

Fruit peel samples were collected at 0, 3, 24, and 72 h after sucrose treatment. Ten fruits were sampled at each time point, and three biological replicates were performed. The peels were sampled and frozen immediately in liquid nitrogen and then transferred to a −80 °C refrigerator for RNA extraction, cDNA library construction, and further physiological analysis.

### 4.2. Measurement of Total Phenol and Anthocyanin Content

The total phenol and anthocyanin were extracted from pear peels treated with or without sucrose at indicated time points, and the measurements were performed as described previously with minor modifications [14]. About 0.2 g of peels were sampled and immediately frozen with liquid nitrogen and ground to powders in a 2 mL tube, and then the mixture was incubated in 1 mL solution of HCl/methanol (1/99, *v*/*v*) in the dark for one hour at 4 °C. The samples were centrifuged at 9500× *g* at 4 °C for 10 min, and then the extract was transferred to a new tube for total phenol and anthocyanin measurement. The absorbances of the extract were recorded at 530 and 600 nm using SpectraMax i3x Multi-Mode Detection Platform (Molecular Devices, Sunnyvale, CA, USA) for anthocyanin determination, and the absorbances were measured at 280 nm for total phenol determination [61]. Five repetitions were performed for each sample.

### 4.3. Library Preparation and RNA Sequencing

The total RNA was isolated from pear peels with a Fast Plant RNA Kit for Polysaccharides & Polyphenolics-Rich according to the manufacturer’s instructions (ZOMANBIO, Beijing, China). The quality of the total RNA was examined by agarose gel electrophoresis and assessed on an Agilent 2100 Bioanalyzer (Agilent Technologies, Palo Alto, CA, USA). The cDNA libraries were constructed by Gene Denovo Biotechnology Co. (Guangzhou, China) and sequenced with the approach of the Illumina Novaseq6000 platform. Raw reads were further filtered according to the method of Chen et al. [62]. The clean reads were mapped to the *P. communis* Bartlett DH Genome (https://www.rosaceae.org/, accessed on 18 November 2021) using HISAT2.2.4 [63].

### 4.4. RNA Isolation and RT-qPCR Analysis

The peels of the ‘Kuerle Xiangli’ were frozen in liquid nitrogen and ground into powder. The total RNA was isolated using an RNA Extraction Kit (ZOMANBIO, Beijing, China) according to the manufacturer’s instructions. Each RNA sample was subjected to DNase I to remove genomic DNA. The cDNA was synthesized according to the protocol of TransScript One-Step gDNA Removal and cDNA Synthesis SuperMix (TransGen Biotech, Beijing, China). TransStart Top Green qPCR SuperMix (TransGen Biotech, Beijing, China) was used for the RT-qPCR analysis, which was performed on Roche LightCycler 480 system (Roche, Basel, Switzerland). The pear *PcTubulin* gene was used as the internal control for RT-qPCR analysis. The relative expression level of each gene was determined by the 2^−ΔΔCt^ method [64]. Each gene expression analysis in this research was repeated three times. The specific primers used for RT-qPCR analysis are listed in Table S4.

### 4.5. DEGs, GO, and KEGG analysis

The expression abundance and variations of each gene were calculated by the FPKM (fragment per kilobase of transcript per million mapped reads) method using RSEM software. The differences in the transcript levels between the two different groups were analyzed using DESeq2 software, and the genes/transcripts were considered as DEGs by |log2 (fold change)| ≥ 1 with the false discovery rate (FDR) value below 0.05. Volcano plots of the DEGs between different groups were performed using the VolcanoPlot function in the R software. Gene ontology (GO) enrichment analysis for the DEGs was conducted based on the GO database (http://www.geneontology.org/, accessed on 10 December 2021). The pathway enrichment analysis for the DEGs was conducted based on the Kyoto Encyclopedia of Genes and Genomes (KEGG) database (https://www.kegg.jp/, accessed on 10 December 2021). The *p*-value of <0.05 was set as the cutoff criterion.

### 4.6. WGCNA and Gene Network Construction

WGCNA was performed using the WGCNA R package (v 1.61, Peter Langfelder, Los Angeles, CA, USA) [65]. A total of 18,898 genes (FPKM > 1) were used for the construction of the WGCNA network. The automatic network construction function block wise was used to build the modules. The hub genes related to anthocyanin biosynthesis in the “MM. skyblue” module was used to construct co-expression networks and visualized the candidate target genes using Cytoscape (v3.5.1, Paul Shannon, Seattle, WA, USA).

### 4.7. Principal Component Analysis

In this research, PCA was performed with R package models (http://www.rproject.org/, accessed on accessed on 20 November 2021) to analyze the correlation of different samples.

### 4.8. Statistical Analysis

Statistical analysis was performed using the software Microsoft Excel 2010. Graphs were obtained from the GraphPad Prism software and Microsoft Excel 2010. The values in each figure were the mean ± SD of three replicates. Significant differences were analyzed using a Student’s *t*-test, and differences at *p* < 0.05 (*) and *p* < 0.01 (**) were labeled for the statistical tests.

### 4.9. Accession Numbers

All raw reading sequences were uploaded in NCBI’s sequence read archive (SRA) under the accession number PRJNA826346.

## 5. Conclusions

The application of 3.0% exogenous sucrose to the pear cultivar ‘Kuerle Xiangli’ promoted anthocyanin accumulation. Sucrose regulates the transcript levels of a range of anthocyanin biosynthesis-related genes, including regulatory genes and structural genes. Sucrose application affects many biological processes including polyphenol synthesis and carbohydrate metabolism, which may contribute to the coloration of pear skin. Collectively, these results indicate that sucrose serves as a basic substrate or signal that acts upstream of the anthocyanin biosynthesis pathway and thus plays an important role in the regulation of fruit coloring in pear.

## Figures and Tables

**Table 1:** Overview of the transcriptome data and quality control of pear peel subjected to sucrose treatment at different time points.

Sample	Raw Data	Clean Data (%)	Q20 (%)	Q30 (%)	GC (%)	Total Mapped (%)
SUC-0 h-1	82,667,734	82,450,850 (99.74%)	98.14%	94.09%	45.72%	71,106,922 (86.29%)
SUC-0 h-2	47,461,574	47,337,338 (99.74%)	98.00%	93.70%	45.57%	40,788,425 (86.21%)
SUC-0 h-3	59,384,286	59,245,816 (99.77%)	98.19%	94.17%	45.73%	51,538,794 (87.05%)
SUC-3 h-1	50,820,492	50,699,094 (99.76%)	98.22%	94.29%	45.76%	44,155,124 (87.15%)
SUC-3 h-2	59,876,342	59,730,970 (99.76%)	98.21%	94.21%	45.77%	51,955,390 (87.05%)
SUC-3 h-3	49,816,612	49,697,046 (99.76%)	98.27%	94.41%	45.82%	42,609,191 (85.85%)
SUC-24 h-1	53,803,782	53,669,030 (99.75%)	98.10%	94.07%	46.13%	46,304,024 (86.34%)
SUC-24 h-2	66,094,172	65,934,960 (99.76%)	98.14%	94.12%	46.15%	56,921,111 (86.44%)
SUC-24 h-3	51,003,520	50,893,952 (99.79%)	98.16%	94.15%	46.18%	44,200,763 (86.97%)
SUC-72 h-1	50,473,534	50,349,396 (99.75%)	98.14%	94.17%	46.52%	43,343,719 (86.31%)
SUC-72 h-2	57,464,526	57,323,698 (99.75%)	98.18%	94.27%	46.20%	49,017,640 (85.89%)
SUC-72 h-3	61,896,448	61,749,868 (99.76%)	98.11%	94.11%	46.21%	53,437,535 (86.70%)

## Data Availability

Not applicable.

## Supplementary Materials

The following supporting information can be downloaded at: https://www.mdpi.com/article/10.3390/genes13061064/s1, Figure S1: Analysis of DEGs in different groups. (A) Volcano plot of DEGs in response to sucrose treatment for 3 h vs. 24 h. (B) Volcano plot of DEGs in response to sucrose treatment for 3 h vs. 72 h. (C) Volcano plot of DEGs in response to sucrose treatment for 24 h vs. 72 h. Red dots indicate upregulated genes, and blue dots indicate downregulated genes. Gray dots indicate no significant differentially expressed genes. Ns, no significant difference. The numbers of upregulated and downregulated DEGs are shown in the corresponding figures; Figure S2: GO enrichment analysis of DEGs in different groups. (A) GO enrichment analysis of the DEGs in response to sucrose treatment for 24 h and 3 h. (B) GO enrichment analysis of the DEGs in response to sucrose treatment for 72 h and 3 h. (C) GO enrichment analysis of the DEGs in response to sucrose treatment for 72 h and 24 h. The dots indicate the number of DEGs, and the color scale indicates the *q*-value; Figure S3: KEGG enrichment analysis of DEGs in different groups. (A) KEGG enrichment analysis of the DEGs in response to sucrose treatment for 24 h and 3 h. (B) KEGG enrichment analysis of the DEGs in response to sucrose treatment for 72 h and 3 h. (C) KEGG enrichment analysis of the DEGs in response to sucrose treatment for 72 h and 24 h; Figure S4: Consistency analysis between RNA-seq data and RT-qPCR results; Figure S5: Measurement of the anthocyanin content in ‘Kuerle Xiangli’ under sucrose, glucose and fructose treatments after 96 h. Asterisks indicate statistical significance (*, *p* < 0.05; and **, *p* < 0.01) calculated by Student’s *t*-test; Table S1: Information of structural genes involved in sucrose-mediated anthocyanin biosynthesis pathways in pear; Table S2: Information of several possible regulatory genes involved in sucrose-mediated anthocyanin biosynthesis in pear; Table S3: Transcript levels and protein sequence of sugar transporter genes in pear subjected to sucrose treatment; Table S4: Primers used for RT-qPCR analysis in the experiment.
