# Supplementary material for: Transcriptome Analysis Reveals Roles of Sucrose in Anthocyanin Accumulation in ‘Kuerle Xiangli’ (Pyrus sinkiangensis Yü)
Source: Genes (Basel). 2022 Jun 14;13(6):1064. doi: 10.3390/genes13061064 (PMC9222499; doi:10.3390/genes13061064)
Supplement: Supplementary file 1 [file genes-13-01064-s001.zip › Supplementary Figures.pdf]

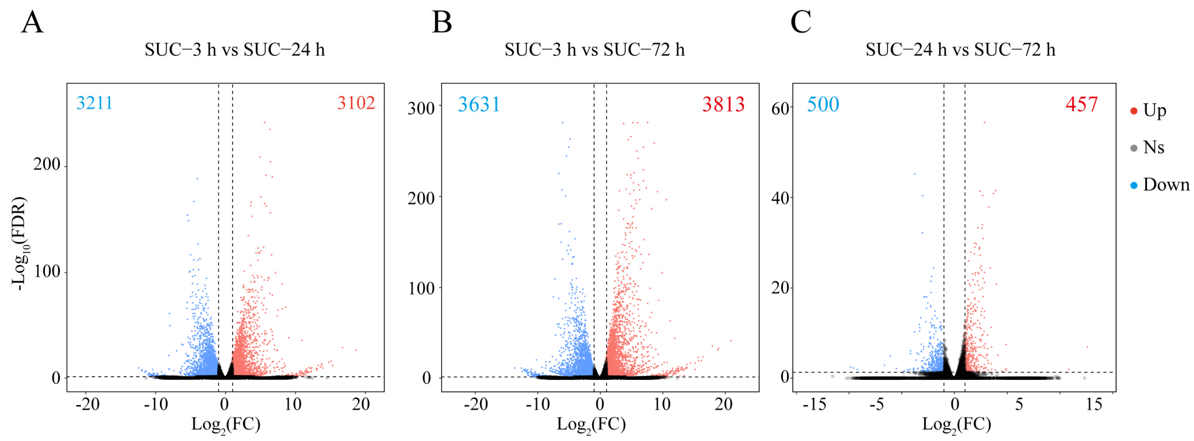

Figure S1. Analysis of DEGs in different groups. (A) Volcano plot of DEGs in response to sucrose treatment for 3 h vs. 24 h. (B) Volcano plot of DEGs in response to sucrose treatment for 3 h vs. 72 h. (C) Volcano plot of DEGs in response to sucrose treatment for 24 h vs. 72 h. Red dots indicate upregulated genes, and blue dots indicate downregulated genes. Gray dots indicate no significant differentially expressed genes. Ns, no significant difference. The numbers of upregulated and downregulated DEGs are shown in the corresponding figures.

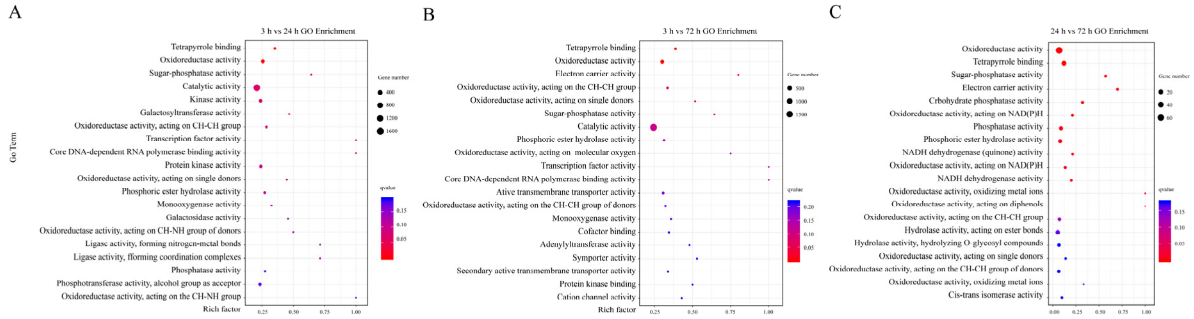

Figure S2. GO enrichment analysis of DEGs in different groups. (A) GO enrichment analysis of the DEGs in response to sucrose treatment for 24 h and 3 h. (B) GO enrichment analysis of the DEGs in response to sucrose treatment for 72 h and 3 h. (C) GO enrichment analysis of the DEGs in response to sucrose treatment for 72 h and 24 h. The dots indicate the number of DEGs, and the color scale indicates the *q*-value.

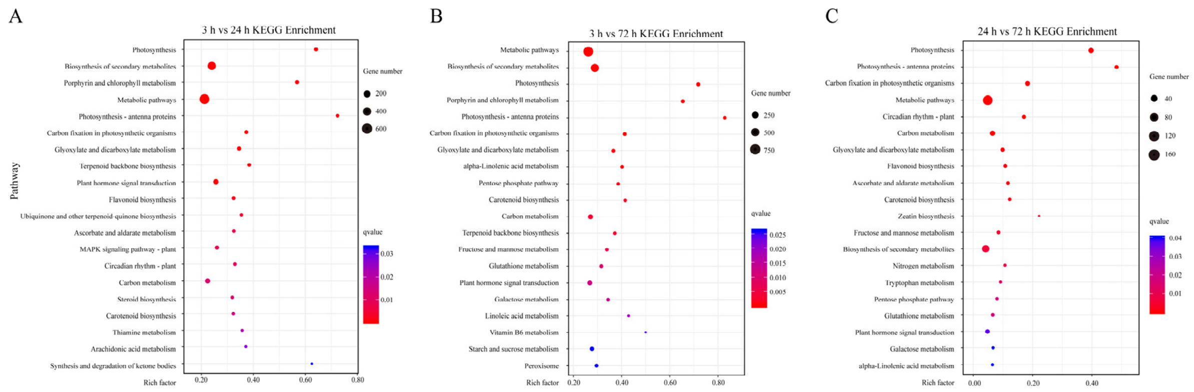

Figure S3. KEGG enrichment analysis of DEGs in different groups. (A) KEGG enrichment analysis of the DEGs in response to sucrose treatment for 24 h and 3 h. (B) KEGG enrichment analysis of the DEGs in response to sucrose treatment for 72 h and 3 h. (C) KEGG enrichment analysis of the DEGs in response to sucrose treatment for 72 h and 24 h.

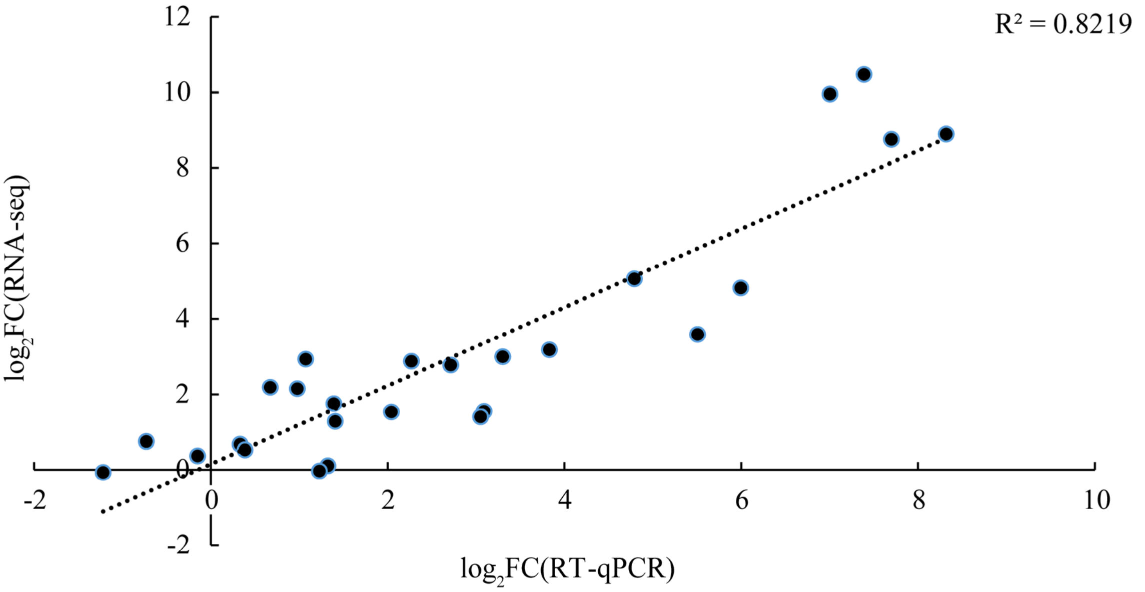

Figure S4. Consistency analysis between RNA-seq data and RT-qPCR results

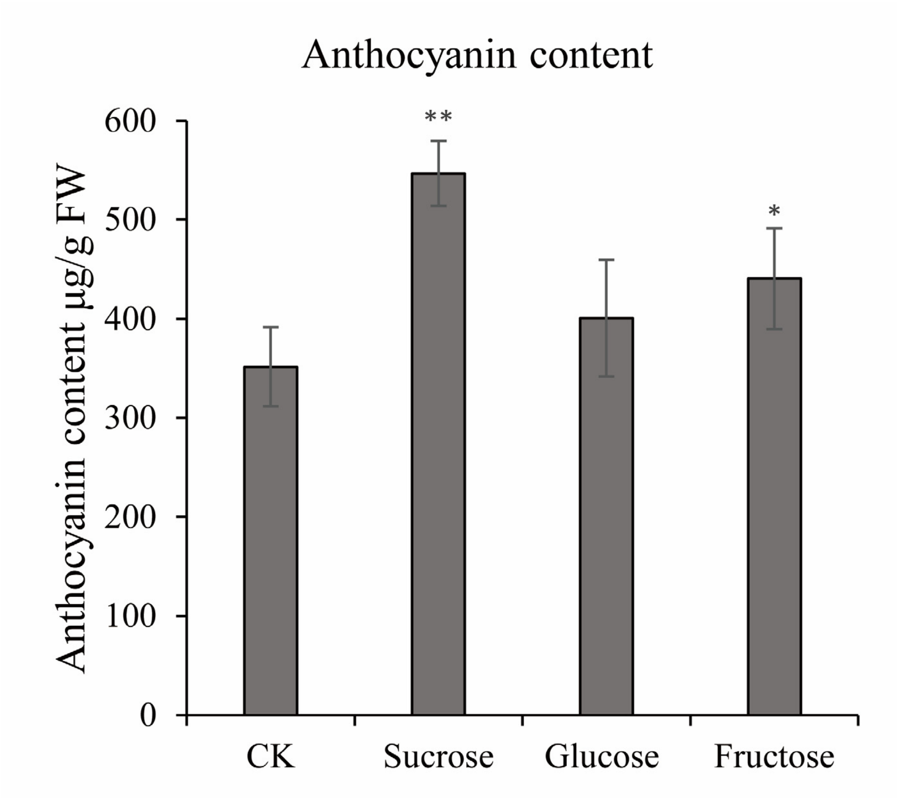

Figure S5. Measurement of the anthocyanin content in 'Kuerle Xiangli' under sucrose, glucose and fructose treatments after 96 hours. Asterisks indicate statistical significance (*, $p < 0.05$; and **, $p < 0.01$) calculated by Student's *t*-test.
